# The Evolving Landscape of Malaria Prevention Strategies: A Review of Recent Developments

**DOI:** 10.3390/pathogens15020137

**Published:** 2026-01-26

**Authors:** Yathavi Charavanamuttu, Akosua Agyeman Wamba, Andrew W. Taylor-Robinson, Temi Lampejo

**Affiliations:** 1 Faculty of Life Sciences and Medicine, King’s College London, London WC2R 2LS, UK; yathavi.charavanamuttu@kcl.ac.uk; 2 Department of Infection Sciences, King’s College Hospital, London SE5 9RS, UK; akosua.wamba@nhs.net; 3 College of Health Sciences, VinUniversity, Hanoi 100000, Vietnam; andrew.tr@vinuni.edu.vn; 4 Center for Global Health, Perelman School of Medicine, University of Pennsylvania, Philadelphia, PA 19104, USA

**Keywords:** malaria prevention, vaccine, monoclonal antibody, chemoprevention, *Plasmodium*

## Abstract

Malaria continues to impose a devastating disease burden globally despite control efforts spanning decades. Its elimination has been hindered by parasite and vector complexity and emerging drug and insecticide resistance, along with unremitting barriers to uptake of preventative strategies largely driven by social inequities, cost constraints, and logistical challenges in implementation. This review synthesises current and emerging prevention strategies, including vector control, chemoprevention and immunoprophylaxis. Insecticide-treated nets and indoor residual spraying remain cornerstones of vector control, although their effectiveness is increasingly compromised by widespread insecticide resistance. Chemoprevention, including intermittent preventive treatment in pregnancy and seasonal malaria chemoprevention in children, has proven highly efficacious, yet uptake remains below WHO targets and concerns about drug resistance remain. Recent advances in vaccines, notably RTS,S/AS01 and R21/Matrix-M, represent landmark achievements, with large-scale rollouts demonstrating reductions in severe disease and mortality. Novel approaches, such as monoclonal antibodies and genetically modified mosquitoes, offer promising avenues for future prevention. However, challenges remain in ensuring equitable access, sustaining efficacy in the face of evolving parasite and vector biology, and integrating interventions into diverse health systems. This review highlights the need for adaptive, multifaceted approaches to achieve malaria elimination goals.

## 1. Introduction

Despite being a highly preventable and treatable disease, malaria continues to impose a devastating public health burden [1]. The World Health Organization (WHO) estimates that in 2024, there were 282 million malaria cases globally, an increase of 1 million from the previous year, and with an estimated 610,000 malaria-associated deaths [1]. The highest and most severe disease burden remains in Africa, accounting for 94% of confirmed malaria cases and 95% of deaths [1].

Malaria is caused by a single-celled intracellular protozoan parasite of the *Plasmodium* genus. Five species are known to be pathogenic to humans: *Plasmodium falciparum*, *P. vivax*, *P. malariae*, *P. ovale* (comprising the two genetically distinct subspecies *P. ovale wallikeri* and *P. ovale curtisi)*, and *P. knowlesi* [2]. *P. falciparum* predominates in sub-Saharan Africa and is responsible for the most severe malaria-associated morbidity and mortality, whereas *P. vivax* is the most geographically widespread species and is most prevalent in South America and Asia [3]. Human infection occurs through the inoculation of *Plasmodium* sporozoites when a female *Anopheles* mosquito takes a blood meal [4]. These sporozoites rapidly migrate to the liver, infect hepatocytes, and undergo asexual replication via exoerythrocytic schizogony (pre-erythrocytic stage). During this liver stage of infection, the human host is usually asymptomatic. In human *P. vivax* and *P. ovale* infections only, the parasite can remain quiescent in the liver in the form of hypnozoites, which can reactivate up to several months later causing relapsing infections. The hepatic phase culminates in the release of merozoites into the peripheral circulation, where they invade erythrocytes and initiate the intraerythrocytic cycle, progressing through trophozoite and schizont stages (erythrocytic stage). This vegetative growth phase corresponds to the symptomatic stage of disease [2,4]. The life cycle is perpetuated when a mosquito ingests circulating gametocytes during a blood meal, facilitating onward parasite transmission upon subsequent human bites.

Malaria usually presents as a non-specific febrile illness with systemic manifestations. Typical symptoms include cyclical fevers, headaches, vomiting, and diarrhoea [2]. Severe disease may be characterised by impaired consciousness, hypoglycaemia, shock, hyperparasitaemia and renal impairment, which can be fatal if untreated [5]. Children under five years of age, pregnant women, migrant populations, and persons with disabilities are at heightened risk of complications and mortality [6]. Although severe malaria is typically associated with *P. falciparum* infection, it has become increasingly recognised that non-falciparum *Plasmodium* species, particularly *P. vivax*, can also exhibit severe, life-threatening clinical manifestations.

Rapid diagnosis and treatment of malaria are crucial but in order to achieve elimination, the ultimate goal of the WHO and global malaria community, preventative measures need to be prioritised, made readily available and effectively implemented on a worldwide scale. Despite targeted prevention measures in reducing mosquito-borne diseases dating back centuries, with several advances in malaria prevention strategies over this time, malaria continues to represent a major global health burden. Progress towards achieving malaria elimination has been hindered by a multitude of factors including the availability of preventative measures in certain resource-limited settings but also insufficient uptake (e.g., chemoprevention) despite availability in some communities due to particular beliefs, misinformation and knowledge gaps surrounding malaria [7]. An additional challenge is the changing epidemiology of malaria, in which ongoing climate change could drive the future establishment of *Anopheles* species in novel geographical areas [8,9]. Locally acquired (autochthonous) human malaria infections have been reported in regions where malaria was previously considered eliminated, including sporadic cases in Spain, Italy and Metropolitan France as well as a local outbreak in Greece (the latter also linked to mass migration) [10,11,12,13,14].

Heightened global efforts with a multi-faceted approach are required to overcome existing barriers to malaria elimination. To date, malaria prevention strategies fall mainly into three domains: vector control, chemoprevention, and immunoprophylaxis. Over the last decade, exciting and promising developments have been achieved, including the introduction of vaccines and the development of monoclonal antibodies (mAbs). These novel immune-based strategies have largely focused on *P. falciparum* malaria, given its disease severity. This review provides an overview of existing and emerging malaria prevention strategies, highlighting current successes, ongoing barriers, and areas for future development.

## 2. Vector Control

The primary aim of vector control methods is to reduce mosquito–human contact. This can be achieved through a combination of physical barriers, chemical deterrents, and wider ecological strategies of vector population control. Vector control methods have been widely implemented in malaria-endemic regions for many years. The techniques themselves have evolved over time in response to emerging challenges including insecticide resistance and environmental changes.

### 2.1. Insecticide-Treated Nets

Insecticide-treated nets (ITNs) are a key intervention for malaria control, serving as both a physical barrier and chemical deterrent [15]. They can be impregnated with a single insecticide (predominantly pyrethroids) or dual active ingredients (containing a secondary insecticide such as piperonyl butoxide) [16].

The use of nets to protect against mosquitoes dates as far back as the 5th century BCE and the subsequent addition of chemical repellents emerged under the demands of World War 2 by both the US (which primarily used dichlorodiphenyltrichloroethane (DDT)) and the USSR (which used juniper oil) [17]. It was, however, not until the 1980s that ITNs were integrated into global health policy and use became more widespread [18]. An earlier systematic review (published in 2004) demonstrated that ITN use in areas of stable transmission reduced episodes of uncomplicated malaria by 50% compared with no nets and by 39% compared with untreated nets, and that an estimated 5.5 lives could be saved per 1000 children protected [19]. In the late 2000s, conventional ITNs were largely replaced by long-lasting insecticidal nets (LLINs; suitable for 3 years of use) [20,21]. This switch was particularly important, as replacing ITNs before expiration of their prescribed lifespan is vital for effective vector control [22]. The impact of LLINs has been substantial; for example, LLIN deployment in Laos reduced malaria incidence by 54.1% between 2016 and 2023 [23]. A meta-analysis in 2018 that investigated a range of malaria prevention measures, including ITNs, untreated nets, prophylactic drugs and insect repellent sprays, found that ITNs were the only preventative strategy with strong evidence for their effectiveness [24]. The efficacy of ITNs has also been shown to be maintained during humanitarian emergencies [25].

Extensive evidence continues to support the use of ITNs as key in malaria prevention, and thus between 2004 and 2024 more than 331 billion ITNs were distributed globally [1]. Utilisation, however, remains suboptimal in several malaria-endemic regions [26,27]. A meta-analysis in Ethiopia reported ITN usage at 56.3%, below the national target of 80% by 2025/26 [26,27]. A recent systematic review of ITN use in sub-Saharan Africa found a similar usage rate of 58.3% [28]. In addition to underusage of ITNs, an additional growing concern with widespread LLIN use is the emergence of insecticide resistance amongst mosquito populations [29]. Transmission models indicate that even low levels of insecticide resistance can sufficiently reduce mosquito mortality whereby significant increases in malaria incidence can occur [30]. Evidence of insecticide resistance has already been documented across multiple *Anopheles* species worldwide. A study conducted across 12 districts of central India reported resistance to DDT and malathion in *An. culicifacies* mosquitoes, alongside emerging resistance to pyrethroids including alphacypermethrin and deltamethrin [31]. In Cameroon, a recent study of pyrethroid resistance reported that all randomly sampled bed nets were deemed ineffective against *An. gambiae*, the predominant local vector [32]. *Anopheles funestus* has similarly developed resistance to pyrethroids, organochlorides, and carbamates [33]. Of note, the expanding prevalence of *An. stephensi* across Africa represents an additional challenge, given its adaptability to urban environments and resistance to multiple insecticide classes [34].

In response to escalating resistance, ITNs incorporating dual active ingredients (DAIs) have been introduced. In 2023, the WHO recommended two new DAI nets: pyrethroid–chlorfenapyr nets, combining a pyrethroid with a pyrrole insecticide, and pyrethroid–pyriproxyfen nets, pairing a pyrethroid with an insect growth regulator [35]. A recent meta-analysis demonstrated high efficacy of these nets, with populations that received DAI ITNs experiencing 305 fewer malaria cases per 1000 person-years compared with pyrethroid-only nets [36]. Notably, DAI ITNs retained superior effectiveness even when torn, outperforming intact pyrethroid-only nets [37].

Despite concerns regarding increasing resistance and a rise in the mosquito populations able to withstand the insecticides used in ITNs, there remains a strong body of evidence demonstrating their benefit in reducing illness and mortality (particularly in children) associated with malaria [38].

### 2.2. Indoor Residual Spraying

Indoor residual spraying (IRS), the regular application of insecticides to household walls, is another vector control strategy. The effects of the chemical insecticides typically last several months; mosquitoes landing on the insecticide-applied walls are killed. A global systematic review and meta-analysis of IRS use demonstrated significantly higher effectiveness against malaria in regions with ≥80% IRS coverage, whereas IRS was found to be far less effective in preventing malaria when IRS coverage was <80% [39]. Overall, they found a 65% reduced risk of malaria by performing IRS and that pyrethroid-based IRS showed the greatest efficacy of the various insecticides evaluated. However, rising resistance amongst vector populations necessitates ongoing surveillance. Interestingly, a systematic review and meta-analysis of IRS use in sub-Saharan Africa found that in communities already using ITNs, the addition of IRS with non-pyrethroid insecticides reduced malaria prevalence whereas pyrethroid-based IRS did not [40]. Although not confirmed, the authors hypothesised that their findings could be due to pyrethroid resistance.

Enhanced efforts are needed to increase IRS coverage in malaria-endemic settings, and despite conflicting evidence on the optimal type of insecticide, their overall use in conjunction with other preventative measures is likely to positively impact upon malaria control. The WHO also highlights that the benefits of IRS extend beyond malaria control, as it can also eliminate *Aedes* spp. mosquitoes responsible for transmitting viral pathogens such as dengue, chikungunya, and yellow fever [6].

### 2.3. Topical Repellents and Insecticides

Topical repellents are considered an important strategy for the prevention of malaria, particularly for travellers to endemic settings, and have demonstrated to be up to 100% effective with appropriate use [41]. For those living in malaria-endemic settings, there is insufficient evidence demonstrating that topical repellents, in the presence of other vector control methods, contributes to a significant reduction in malaria transmission [42,43]. However, a Cochrane review, albeit based on a small body of evidence (four randomised controlled trials, of which two were conducted in refugee camps), reported that in high-risk populations, such as refugees who may not have access to standard vector control methods, topical repellents may slightly reduce *P. falciparum* prevalence [42]. A large Cambodian study found that free mass distribution of topical repellents did not reduce malaria transmission although this was largely attributed to human behaviour (inconsistent daily compliance or appropriate use of the repellents) [44]. Nevertheless, topical repellents are considered safe with a low rates of adverse effects (0.6%), which are generally limited to mild cutaneous reactions [42].

Randomised controlled trials have demonstrated the efficacy (providing complete protection from *Anopheles* mosquitoes for up to 6 h) of three main topical repellent active agents, N,N-Diethyl-*meta*-toluamide (DEET), *p*-Menthane-3,8-diol (PMD) and Icaridin [45,46,47]. These compounds work by either stimulating avoidance behaviour or modulating odorant receptor protein activity [48]. Aside from inappropriate use (such as ingestion or eye exposure), DEET and Icaridin usage is not associated with severe adverse effects but there is inadequate evidence from human trials to conclude the same level of safety in PMD usage [41,49]. However, all three repellents have been deemed safe for use by the WHO and the United States Environmental Protection Agency (USEPA). DEET has also been shown to be safe in pregnancy, breastfeeding and in children over 2 months of age [41,50,51]. Research is also being undertaken to identify additional novel repellents; the compound 1-allyloxy-4-propoxybenzene 3c{3,6} has shown particular early promise but is yet to progress to human clinical studies [52].

Overall, topical repellents have a long history of serving as a useful tool against mosquito bite prevention with a superb safety profile, but the required frequency of application of topical repellents hinders their effectiveness [53]. They continue to have an important role in malaria prevention and can be highly effective with correct and consistent use. There is a need for new repellents in view of changing vector behaviour, and the availability of longer-lasting repellents requiring less frequent application would be a welcome addition to the repertoire of available topical repellents.

An additional emerging use of topical repellents/insecticides is in baby wraps treated with insecticides to protect babies/young children against malaria. Insecticide-treated clothing has been used for many years, primarily by the military but also in certain recreational activities, for protection against a range of arthropods including mosquitoes [54]. Permethrin is the most commonly used active ingredient but several others including DEET, cyfluthrin, bifenthrin and deltamethrin have been trialled. Insecticide-treated clothing has been reported to provide between 0% and 75% protection against malaria [54]. A recent double-blind randomised controlled trial in Uganda found that permethrin-treated baby wraps significantly reduced the incidence of clinical malaria (0.73 cases per 100 person-weeks in the intervention group compared to 2.14 in the control group) [55].

## 3. Environmental Control

Environmental control methods are an additional avenue through which mosquito–human contact and malaria transmission may be reduced. One such method is larviciding: the application of insecticides to bodies of water, or water containers, with the intention to kill the aquatic immature larvae and pupae forms of mosquitoes [56]. The WHO has recommended larviciding as a supplement to existing standard methods of vector control [57]. Larviciding typically forms part of a large-scale programme, usually taking place on common land and/or water, performed by environmental control officers employed by the local government or public health authorities. There is strong evidence for larviciding in areas where a sufficient proportion of larval habitats can be targeted [56,58,59]. Two randomised controlled trials in Sri Lanka reported a four-fold reduction in malaria incidence and a Cochrane review evaluating the effectiveness of mosquito larval source management for preventing malaria demonstrated a three-fold reduction in parasite prevalence [59,60,61]. However, in rural areas with extensive larval habitats, the evidence for larviciding is less convincing [56,59].

Larviciding seems to be most effective in urban or seasonal settings where larval habitats are limited and accessible, but its implementation remains complex, requiring ecological expertise and robust monitoring frameworks. Access to WHO-prequalified products is restricted, and high costs deter scale-up, although innovations such as drone-based delivery and regional collaboration platforms may enhance feasibility and impact [57].

Other methods of environmental vector control include predation by dragonflies and damselflies (both of which are natural predators of mosquitoes), which has been demonstrated to be effective, and habitat manipulation (such as home modifications to reduce mosquito entry points), for which the evidence is conflicting [62,63].

## 4. Genetic Modification of the Vector

Advances in gene-based technology, such as the progression of the CRISPR/Cas9 technique (considered a highly effective gene-editing tool), have facilitated the development of genetically modified mosquitoes (GMMs) [64]. These mosquitoes are engineered to become incapable of transmitting specific pathogens and therefore rendered refractory vectors. Strategies include engineering mosquitoes to express exogenous anti-pathogen factors or knocking out host factors essential for pathogen development. Where utilised, the intention would be for released GMMs to progressively replace wild-type populations. This process has already been implemented in parts of Brazil, the Cayman Islands, Panama and India, targeting *Ae. aegypti*, a mosquito species that transmits arboviruses such as dengue, zika and chikungunya viruses. Since 2019, more than one billion GMMs have been released [65]. There are no perceived adverse risks to humans, animals or the environment although longer-term data on their effectiveness, safety and environmental impact are awaited.

In aiming to curb malaria transmission there is potential for this technology to be implemented in *Anopheles* mosquito populations. For example, mosquitoes engineered to express human PAI-1 (huPAI-1), a serine protease inhibitor that inhibits fibrinolysis by preventing the conversion of human plasminogen to plasmin in the midgut, have been shown to inhibit the sexual stages of the *Plasmodium* life cycle, and kill sporozoites, thereby reducing transmission [66]. In May 2024, the first pilot release of genetically engineered *An. stephensi* began in Djibouti, and if proven successful, further large field trials are expected to follow [67].

GMMs are considered an ecological method of vector control, as their release does not involve chemical contamination of the environment, in the form of insecticides. However, there are ethical questions to address, and an explicit need to carefully consider factors such as potential non-target effects and unintentional pathogen virulence; for example, the *An. gambiae* complex can transmit seven other human and animal pathogens in addition to malaria [68,69]. Moreover, parasite virulence may evolve in response to altered transmission dynamics [69].

An additional research avenue is the leveraging of mosquito-associated bacteria to render the mosquitoes refractory to transmission. For example, the *Delftia tsuruhatensis* TC1 symbiont, which is found in some *Anopheles* mosquitoes, and *Wolbachia pipientis* have both shown potential to reduce transmission through inhibiting the early stages of *Plasmodium* development and subsequent transmission by *Anopheles* mosquitoes [70,71,72].

Overall, gene-based approaches represent a novel and exciting ecological strategy to reduce malaria transmission without reliance on insecticides, although careful evaluation of ecological consequences, pathogen adaptation, and ethical considerations remains essential. If proven safe and effective, these technologies could complement existing vector control measures, expanding the malaria prevention toolbox with sustainable, non-chemical interventions.

## 5. Chemoprotection

Antimalarial drugs are commonly used by travellers visiting malaria-endemic areas. However, they are not widely deployed as a standard intervention for populations local to these settings [73], for whom for several years the WHO has advised targeted use rather than widespread deployment. The vast majority of malaria chemoprotection studies and initiatives have focused on *P. falciparum*, with limited data on *P. vivax* [74].

### 5.1. Chemoprevention in Pregnancy

The first such recommendation of targeted chemoprotection was in 1988 for pregnant women [73]. The WHO has since targeted an 80% uptake of intermittent preventative treatment in pregnancy (IPTp) in malaria-endemic locations [6]. Yet in 2023 only 67% of those eligible received their first dose, and only 44% received their recommended third dose [6]. While this is an increase from previous years, it remains well below the WHO target. An initial concern regarding IPTp deployment was the potential to promote drug resistance, particularly with suboptimal compliance. However, despite IPTp with sulfadoxine-pyrimethamine (SP; a commonly used agent for malaria chemoprevention) being linked with low to moderate antifolate resistance mutations, there is little evidence that parasite exposure selects for key drug resistance-associated mutations [75].

### 5.2. Seasonal Malaria Chemoprevention

The WHO also advises seasonal malaria chemoprevention (SMC) (giving anti-malarial drugs to children aged 3 months to 5 years on a monthly basis during the peak transmission season) in areas with high seasonal variation in malaria burden [6,76]. SMC has expanded to 19 countries across the African Sahel region and other seasonal transmission zones in sub-Saharan Africa, reaching 53 million children in 2023 [6]. Despite heightened efforts, uptake remains below target in some countries [6]. A study in Ghana identified school attendance, distance from distribution sites, and side-effects as key barriers to adherence [77]. Over 60% of malaria cases in the Sahel region of Africa occur during a 3–4-month annual window and SMC has been proven effective in reducing seasonal malaria transmission during this period in various clinical trials and case–control studies [78,79,80]. The Sahel region, however, has low prevalence of SP resistance. Trials are ongoing in southern Africa, where SP resistance is more prevalent, to establish if SMC could be efficacious [80]. A trial in the Karamoja subregion of Uganda, East Africa, found that using SP plus amodiaquine (SPAQ) and dihydroartemisinin–piperaquine for SMC reduced malaria risk by 94% and 96%, respectively, with no reported concerns regarding the emergence of resistance [81]. In a study assessing the effectiveness of SMC in children under 5 years living in the Savannah region of Ghana, SPAQ reduced malaria-related morbidity by 17% and mortality by 67% [77]. Although these findings are promising, evolving parasite susceptibility may mean that the suitability of particular chemoprotective agents may vary regionally and over time [82].

### 5.3. Additional Chemoprevention Measures in Children

The WHO has also issued conditional recommendations for additional chemoprotection strategies [83]. Intermittent preventive treatment in school-aged children (IPTsc) may reduce transmission and adverse outcomes, although evidence demonstrating its effectiveness in reducing the prevalence of malaria remains limited [84]. Another conditional recommendation is post-discharge malaria chemoprevention (PDMC) for children admitted with severe anaemia [83]. Administration of PDMC has been associated with a 77% reduction in mortality and 55% reduction in all-cause readmissions, although protection is not long-lasting [85].

Perennial malaria chemoprevention (PMC) is a newly formulated strategy endorsed by the WHO in 2022 that aims to protect children up to 24 months of age in areas with high rates of malaria transmission [83]. A Ghanaian study found that SP-based PMC in infants provided protection lasting approximately 42 days following treatment [86]. Another study of SP-based PMC in Tanzanian infants, an area of high antifolate resistance, found that SP provided some protection only in the first month after treatment (protective efficacy of 64.5%) but not for extended periods of time [87]. No protection against clinical malaria of the short-acting antimalarial chlorproguanil-dapsone was reported. However, the investigators did find that long-acting mefloquine provided a protective efficacy of 73.3% after 2 months. Interestingly, early-stage clinical trials are underway to evaluate MMV371, a long-acting injectable that is metabolised to atovaquone, a common chemoprotective agent [88]. First-in-human trials approved in 2024 aim to determine whether sustained drug levels can reduce dosing frequency compared with existing oral regimens.

### 5.4. Mass Drug Administration

Mass drug administration (MDA) remains contentious. A 2024 meta-analysis showed that MDA reduced incidence and parasitaemia prevalence for up to three months, but protection was not sustained [89]. A recent cluster-randomised trial in Kwale, Kenya, demonstrated that three monthly doses of ivermectin in children aged 5–15 years reduced malaria incidence by 26% compared to controls who received albendazole [90]. Overall, the WHO considers evidence for MDA low-certainty and does not recommend its use in moderate to high *P. falciparum* transmission settings [83].

### 5.5. Challenges in Chemoprevention

Concerns persist regarding drug resistance related to the wider use of chemoprotection. Current evidence suggests that SP-based chemoprotection does not lead to meaningful levels of drug resistance, in part due to the drugs deployed in these interventions differing from those used for treatment [73,75,91]. However, development of rapid resistance has been reported with piperaquine- and pyrimethamine-based therapies [92,93]. Additional concerns include the “rebound phenomenon”, whereby natural immunity may be compromised after cessation of chemoprotection, although evidence to support this notion is weak [73]. Cost and logistical challenges also pose barriers, yet high coverage in SMC rollouts and the availability of inexpensive antimalarials have improved feasibility [73,94,95]. Despite the discussed challenges, IPTp and SMC remain highly efficacious and cost-effective interventions, delivering substantial reductions in morbidity and mortality in the face of persistent gaps in coverage [94].

### 5.6. Chemoprevention Against P. vivax Malaria

Relative protection of infants against *P. vivax* infection when given routine intermittent preventive treatment with SPAQ has been demonstrated in Papua New Guinea, and mass chemoprevention with primaquine (an oral 8-aminoquinoline derivative) prior to the *P. vivax* transmission season was shown to be efficacious in a study conducted in North Korea [74,96,97]. However, further research is needed to determine the efficacy of, and optimal strategies for, chemoprevention against *P. vivax* malaria. A recent WHO-commissioned systematic review on mass relapse prevention (MRP) to reduce transmission of *P. vivax* found that the oral 8-aminoquinoline agent primaquine, given as a single round over 14 days at a dose of 0.25 mg/kg/day, significantly reduced the incidence of *P. vivax* infections 1 to 3 months after the treatment round in villages that received MRP compared to those that did not (pooled rate ratio [RR] 0.08, 95% CI 0.07–0.08) [98]. Additionally, at 4 to 12 months post-treatment the prevalence of *P. vivax* infection was significantly lower in MRP villages compared to non-MRP villages (odds ratio 0.12, 95% CI 0.03–0.52). No severe adverse events were found. However, no major conclusions could be drawn from this work as only two studies (both non-randomised) were included in the systematic review; one from North Korea (391,357 participants) and the other from Azerbaijan (~30,000 participants). The impact of MDA of tafenoquine, also an oral 8-aminoquinoline derivative but with an extended half-life up to 15 days (therefore longer-acting than primaquine), is being investigated on the Myanmar–Thailand border, where there is consistently a high incidence of *P. vivax* (ClinicalTrials.gov identifier number NCT06575647). Currently, data are lacking to make recommendations regarding the use of chemoprotection to combat *P. vivax* malaria and further studies are awaited.

## 6. Vaccines

Vaccination is a recent but long-awaited addition to the repertoire of available strategies for the prevention of malaria. The first large-scale in-human pilots of a malaria vaccine (the WHO Malaria Vaccine Implementation Programme [MVIP]) began only in 2019 [99]. The extended delay in development has primarily been driven by the complexity of *Plasmodium* parasites and their multi-stage course of infection within the human host, which lends itself to immune evasion. Additionally, the ability of the *Plasmodium* parasite to rapidly evolve, leading to phenotypic antigenic variation, continues to pose challenges to vaccine development. Within the *Plasmodium* life cycle, there are three key stages at which to direct malaria vaccines and mAbs: the pre-erythrocytic, asexual erythrocytic, and sexual erythrocytic stages [100]. The pre-erythrocytic stage, in which there are a relatively small number of sporozoites, serves as the most attractive target for vaccines and mAbs when compared to the erythrocytic stages of infection, the latter comprising potentially millions of parasites circulating in the peripheral blood.

**Figure 1:**
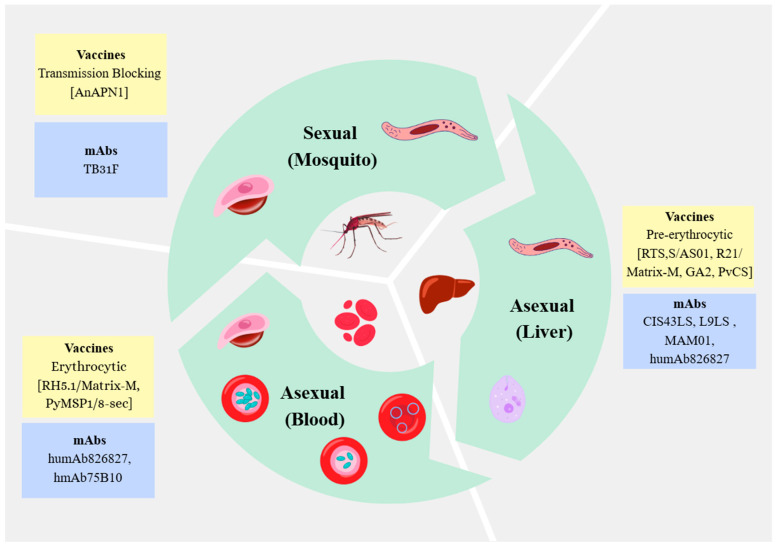
The life cycle of the *Plasmodium* parasite is depicted across three major stages: sexual (mosquito); asexual (liver); and asexual (blood). Vaccine and monoclonal antibody (mAb) strategies are mapped to each stage, reflecting their mechanistic targets and intended points of intervention. Pre-erythrocytic vaccines (e.g., RTS,S/AS01, R21/Matrix-M) and mAbs (e.g., CIS43LS, L9LS) aim to neutralise sporozoites, thereby preventing liver-stage infection and downstream blood-stage parasitaemia. Blood-stage vaccines (e.g., RH5.1/Matrix-M) and mAbs (e.g., humAb826, humAb827) target merozoites during erythrocytic replication, seeking to reduce parasite burden and clinical severity. Transmission-blocking interventions (e.g., AnAPN1 vaccine, TB31F mAb) are directed at inhibiting sexual reproduction within the mosquito midgut, with the goal of halting parasite development and onward transmission. This figure was created using the Canva Pro software package (https://www.canva.com/pro/ (accessed on 2 January 2026)).

Figure 1 schematically illustrates the malaria life cycle, highlighting stages that are targeted by various malaria vaccines and mAbs.

### 6.1. P. falciparum Pre-Erythrocytic Vaccines

The first available *P. falciparum* malaria vaccine, recommended by the WHO in October 2021, was RTS,S/AS01. It is a recombinant protein-based subunit vaccine containing the *P. falciparum* circumsporozoite protein (PfCSP) expressed by sporozoites in the early pre-erythrocytic stage. It is recommended as a four-dose schedule for children 5 months of age. A trial conducted across seven countries in sub-Saharan Africa found the efficacy of the vaccine against all episodes of clinical malaria to be 39%, and 29% against severe malaria over a 4-year follow-up period in children aged 5 to 17 months [101]. Although the vaccine demonstrated a favourable safety profile overall, meningitis, cerebral malaria and female all cause-mortality were notable safety signals requiring further evaluation and monitoring [101,102]. A meta-analysis of randomised controlled trials found rates of severe adverse events (SAEs) comparable between RTS,S/AS01 recipients and rabies vaccine controls, with febrile convulsions and pneumonia amongst the most common SAEs (26.2% in the control group vs. 21.4% in the RTS,S/AS01 group) [103]. In 2023, the WHO also recommended the *P. falciparum* malaria R21/Matrix vaccine, which similarly acts to elicits immune responses against PfCSP, so therefore also a pre-erythrocytic (subunit) vaccine. In children aged 5 to 17 months living in Burkina Faso, this vaccine demonstrated an efficacy of up to 78% in the high-dose adjuvant group following a three-dose primary vaccination course and a booster dose at 12 months [104]. The vaccine was well tolerated and had a favourable safety profile.

The WHO currently recommends either the RTS,S/AS01 or the R21/Matrix-M vaccinations for children living in areas endemic for *P. falciparum* [99]. Children, especially those under the age of 5 years, are known to be particularly vulnerable to severe infection and at higher risk of mortality, due to their immature immune system [3]. Both vaccines are considered easily scalable and cost-effective, hence the wide-scale rollout observed since their introduction [105]. As part of the WHO-led MVIP, more than 2 million children in Ghana, Kenya and Malawi received a malaria vaccine from 2019 to 2023, which led to a 13% attributable drop in mortality and a significant reduction in hospitalisations from severe malaria [99]. As of December 2025, 24 countries in Africa have adopted a vaccination rollout scheme, with plans for expansion coinciding with a dramatic reduction in vaccine cost, supported by equitable pricing agreements with Gavi and UNICEF [106].

Resistance to these pre-erythrocytic *P. falciparum* vaccines has not been an issue thus far but parasite evolution over time does raise concerns regarding its potential future emergence. In theory, combining pre-erythrocytic vaccines with another vaccine targeting a different stage of the parasite life cycle may reduce this risk. The WHO has set a strategic goal to develop by 2030 a multistage *P. falciparum* malaria vaccine that aims to reduce both clinical disease and transmission [105]. One multistage *P. falciparum* vaccine consisting of R21 (targeting the pre-erythrocytic stage) in combination with RH5.1 and R78C (targeting the blood stage) is currently being evaluated in parallel phase 1b and phase 2b trials in infants [105,107].

### 6.2. P. falciparum Whole-Sporozoite Vaccines

Whole-sporozoite vaccines represent an alternative novel method of pre-erythrocytic vaccination [105]. These vaccines use live genetically attenuated *P. falciparum* sporozoites to confer lasting protection. These parasites are typically a single-gene knockout that are designed to arrest development at the late liver stage, thereby preventing release of merozoites into the bloodstream; i.e., they do not progress to the symptomatic erythrocytic stage of *P. falciparum* infection [108]. Recent phase 1 and 2a trials (of controlled human malaria infection) have demonstrated efficacy and safety of a new *P. falciparum* GA2 next-generation whole-sporozoite vaccine [109,110]. However, production techniques are proving difficult to scale up and further larger trials are required to confirm the promising preliminary findings.

### 6.3. P. falciparum Erythrocytic Vaccines

In addition to the significant advances made with pre-erythrocytic *P. falciparum* malaria vaccines, there has also been recent progress in developing erythrocytic vaccines, although there are challenges with antigen selection due to polymorphic variants [105,111]. A delayed third-dose regimen of the erythrocytic RH5.1/Matrix-M *P. falciparum* vaccine (targeting the reticulocyte-binding protein homologue 5; RH5) was shown to be safe and demonstrated 55% efficacy against incidence of clinical malaria 14 days to 3 months post-third vaccination dose in a phase 2b trial of *P. falciparum* malaria in children in Burkina Faso [112].

### 6.4. P. vivax Vaccines

Malaria vaccine efforts to date have understandably focused on *P. falciparum* in view of it being the malaria species responsible for most severe cases and deaths. Additionally, the higher genetic diversity and more complex life cycle of *P. vivax* compared to *P. falciparum* has made development of vaccines targeting *P. vivax* extremely challenging [113,114]. The fact that *P. vivax* infection is often deemed a relatively mild form of malaria has also meant that it has been relatively neglected in terms of vaccination and other preventative strategies. A very small number of candidate *P. vivax* (pre-erythrocytic, erythrocytic and transmission-blocking) vaccines have entered phase I/II clinical studies but have demonstrated poor immunogenicity and lacked protective efficacy [115,116,117,118,119,120]. Moreover, immunity against *P. vivax* is less well understood. Most *P. vivax* antigen candidates for vaccines are polymorphic and induce strain-variable heterogenous immune responses, so thus provide only partial protection [114]. Possibly the most promising *P. vivax* vaccine candidate from clinical trials to date contains a synthetic peptide from the *P. vivax* circumsporozoite protein (PvCSP) formulated with Montanide ISA-51 as an adjuvant (i.e., a pre-erythrocytic vaccine) [114,117]. In a randomised, double-blind, controlled trial of the synthetic PvCSP vaccine, 35 participants were enrolled: 17 malaria-naïve individuals (phase IIa) and 18 “semi-immune” subjects (phase IIb) [117]. Immunisations were administered at months 0, 2, and 6 with either adjuvanted PvCSP or placebo (adjuvant alone). Three months after the third dose, all participants were subjected to human-controlled *P. vivax* infection. Volunteers were considered protected if they did not develop >100 parasites/µL over the 60-day follow-up, termed “sterile protection”. The vaccine was safe and well tolerated. In the phase IIa study consisting of healthy malaria-naïve volunteers, the vaccine demonstrated an overall protective efficacy of 54.5%. However, in semi-immune individuals, no statistically significant difference in sterile protection was found between vaccinated and control groups, so therefore the impact in this population remains uncertain. Larger trials, including those assessing outcomes following natural infection, are required to establish efficacy more clearly. The recurrent and relapsing nature of *P. vivax* infection does, however, make this particularly demanding.

### 6.5. Bivalent Vaccines for Co-Endemic Regions

There are several countries, such as Indonesia, Bangladesh and Ethiopia, where non-falciparum malaria species exist alongside *P. falciparum*, while *P. vivax* is the predominant parasite outside of the sub-Saharan region [3,113,121,122,123]. As *P. falciparum* and *P. vivax* are considered the two most clinically significant malaria species, vaccines providing cross-protection against both parasites in co-endemic regions would also be welcomed. Recently, the efficacy in a pre-clinical model of a novel bivalent vaccine against *P. falciparum* and *P. vivax* expressing multiple antigens including circumsporozoite proteins (PfCSP and PvCSP) and ookinete surface proteins (PfS25 and Pvs25) was demonstrated [124]. It remains to be seen whether this or other bivalent vaccines progress to human trials.

### 6.6. Transmission-Blocking Vaccines

In terms of alternative malaria vaccine targets, those against sexual stages of the parasite life cycle, thereby acting as transmission-blocking vaccines (TBVs), have also been considered [68]. However, progress has been slow due to various factors including a reliance on herd immunity to reduce mosquito infections and prevent transmission [68]. There are, however, various current studies investigating the safety and efficacy of TBVs developed against *P. falciparum* and/or *P. vivax*. A phase 1 double-blind randomised study of a pan-malaria TBV, named AnAPN1, is underway in Gabon [125]. Given the lack of approved vaccines specifically for *P. vivax* malaria, the findings of this and other studies on TBVs in the prevention of non-falciparum malaria, particularly that caused by *P. vivax*, are eagerly awaited [126]. Of further note is that mRNA vaccines are also being explored as a possible preventative strategy for malaria. In preclinical studies in a *P. yoelii* murine model (a rodent parasite which does not naturally infect humans), an mRNA vaccine (PyMSP1/8-sec) has shown promise, opening the door to potential utilisation of mRNA technology in future human malaria vaccines [127].

### 6.7. Combining Vaccines with Chemoprevention

Tackling malaria undoubtedly requires extensive measures beyond simply achieving an effective vaccine. The WHO recommends offering vaccines alongside other preventative measures, including chemoprevention [83]. In order to mitigate the challenge of suboptimal efficacy amongst current vaccines, the possibility of combining pre-erythrocytic vaccines with existing chemoprotection regimes is being explored [111]. A large, randomised control trial demonstrated that administering RTS,S/AS01E with sulfadoxine-pyrimethamine and amodiaquine resulted in a substantially lower incidence of uncomplicated malaria, severe malaria, and deaths from malaria than either chemoprevention or vaccination alone [128]. Longer-term efficacy data, studies in a range of populations and settings, evaluation of differences in host responses to the vaccine, and the possibility of vaccine escape (where the pathogen variant is able to evade vaccine-induced immune responses) are all important considerations to monitor in future with continued and more widespread adoption of malaria vaccines.

### 6.8. Vaccine Uptake and Acceptance

In addition to availability, efficacy and safety, vaccine acceptance is key to the successful implementation and maintenance of malaria vaccination programmes. Across endemic regions of Africa, public perceptions of malaria vaccines vary widely, with willingness to receive vaccinations ranging from as low as 32.3% in Ethiopia to as high as 96% in Sierra Leone, with willingness varying with vaccine type [129]. A meta-analysis identified the aggregate malaria vaccine acceptance rate for children under the age of five in low- and middle-income countries to be 95.3% overall and 94.4% amongst mothers [130]. High perceived susceptibility was a driving factor behind willingness to be vaccinated, with, conversely, low socioeconomic status and the young adult demographic correlating with reduced acceptance [131]. However, both reviews noted heterogeneity in results and potential publication bias, calling for further studies and highlighting the need for context-specific strategies [131,132]. Ongoing efforts are required in malaria-endemic settings to facilitate vaccine uptake, particularly amongst vulnerable community members, in order to make progress towards the ultimate goal of elimination.

Malaria vaccine development has advanced considerably in recent years, with RTS,S/AS01 and R21/Matrix-M now recommended for children in endemic regions, both demonstrating good protective efficacy and favourable safety profiles. Additional approaches, including whole-sporozoite, erythrocytic, transmission-blocking, and multistage vaccines, are under investigation, although challenges remain with antigenic diversity, durability of protection, and particularly limited progress against *P. vivax*. Evidence also supports combining vaccination with chemoprevention to enhance effectiveness. While uptake has been encouraging in many settings, variability in community acceptance highlights the need for context-specific implementation strategies. Overall, currently available vaccines provide a meaningful public health benefit but further research is required to monitor and enhance efficacy, broaden species coverage, and ensure sustained impact. Although these vaccines are primarily targeted at children (a group who suffer a disproportionally high disease burden), future vaccine studies need to consider a range of other vulnerable groups such as pregnant women, travellers and populations at risk of resurgence.

## 7. Monoclonal Antibodies

Passive immunisation, achieved through administration of mAbs, is an emerging tool in malaria prevention [133]. The importance of humoral immunity in malaria is well recognised, with the use of immunoglobulins to treat malaria going back as far as the early 1960s when purified immunoglobulins obtained from malaria-exposed adults were administered to children, leading to a reduction in parasitaemia [134]. Since then, scientific advances have enabled the development of longer-lasting, more potent mAbs. These include modifications to the Fc effector function, multi-specific antibodies and multi-valent antibodies [135]. mAbs targeted against specific pathogens typically have minimal adverse effects due to their higher specificity and ‘natural’ source of origin [100]. Furthermore, they can be produced en masse in bioreactors, making them an attractive option for a disease such as malaria that carries such a major global burden. A major barrier, however, is the high development costs.

### 7.1. P. falciparum Pre-Erythrocytic mAbs

Various mAbs are currently in development, targeting different stages of the *P. falciparum* life cycle [136]. The first to be studied was CIS43LS, developed from antibodies isolated in a patient immunised with a *P. falciparum* whole-sporozite vaccine [137]. A pre-erythrocytic mAb, CIS43LS, targets a highly conserved region of PfCSP. A two-part phase 1 trial, published in 2021, identified the mAb to be both safe and efficacious [137]. None of the nine participants who trialled CIS43LS developed parasitaemia (assessed by detection of *P. falciparum* DNA by polymerase chain reaction [PCR] over a 21-day period following controlled *P. falciparum* infection), compared to five of six controls. CIS43LS was shown to have a serum half-life of 56 days. In a subsequent three-part phase 1, adaptive clinical trial, no serious adverse events were reported, and 82% of participants receiving any dosing regimen remained parasite-free (absence of detectable *P. falciparum* DNA in blood) compared to 100% of controls [138]. No parasitaemia was observed following intravenous or subcutaneous dosing at 5 or 10 mg/kg, whereas all six controls and four of seven participants receiving 1 mg/kg intravenously developed infection [138]. Two phase 2 trials have since evaluated CIS43LS. In Mali, a single 40 mg/kg intravenous dose achieved 88.2% efficacy, while 10 mg/kg achieved 75.0%, each compared to a placebo over a six-month malaria season [139]. This study demonstrated the potential for CIS43LS (40 mg/kg) to meet the WHO target for a single dose of at least 80% efficacy after 3–4 months [139]. A second randomised phase 2 double-blind trial of a single dose of CIS43LS to 330 healthy Malian adults demonstrated 87.4% efficacy (at the higher 40 mg/kg dose) against *P. falciparum* (detected by PCR) over a 6-month malaria season, highlighting the potential of CIS43LS to reduce both disease burden and transmission during peak seasons [140].

L9LS, a next-generation mAb that also targets PfCSP, has shown approximately three-fold greater potency than CIS43 in preclinical studies [100,141]. A phase 1 clinical trial identified no safety concerns, a half-life of 56 days (similar to CIS43LS), and 88% protection against controlled human *P. falciparum* malaria exposure [141]. No parasitaemia was noted in patients who received 5 mg/kg or 20 mg/kg of intravenous L9LS, although parasitaemia was detected in one individual who received a 1 mg/kg intravenous dose, and another individual who received 5 mg/kg subcutaneously [141]. A subsequent phase 2 clinical trial conducted in children 6 to 10 years old in Mali over a 6-month transmission season also identified no safety concerns [142]. Subcutaneous administration was used at 150 mg and 300 mg single doses, achieving 67% and 77% efficacy against clinical malaria, respectively [142]. Results are awaited from another phase 2 study conducted in Kenyan children to assess the protective efficacy of one or two doses of subcutaneously administered L9LS against natural *P. falciparum* challenge [143].

Another PfCSP-targeting mAb has recently been developed following isolation of antibodies from 45 RTS,S/AS01 vaccinees [144]. The variable domains of the selected antibodies were engineered to facilitate large-scale, low-cost manufacturing, leading to the development of the mAb MAM01 [144]. A phase 1 clinical trial in the US found that a single dose of MAM01 (high dose of 40 mg/kg administered intravenously) was effective at preventing *P. falciparum* parasitaemia following controlled exposure (via the bites of five *P. falciparum* NF54 strain-carrying mosquitoes) for 18–26 weeks after MAM01 administration, but not at lower doses [145]. No serious adverse effects were noted.

### 7.2. P. falciparum Transmission-Blocking mAbs

The humanised mAb TB31F was recently demonstrated to be a potentially effective *P. falciparum* transmission-blocking agent. TB31F targets a highly conserved region of the Pfs48/45 protein expressed by *P. falciparum* gametocytes [146]. Pfs48/45 and another protein, Pfs230, are leading candidate *P. falciparum* malaria vaccine targets [100]. *P. falciparum* gametocytes express both proteins during gametogenesis within the human host and thus play a key role in male gamete fertility. Antibodies directed against Pfs48/45 and Pfs230 can inhibit sexual reproduction within the midgut of an infected *Anopheles* mosquito. TB31F therefore targets a different part of the *P. falciparum* life cycle to CIS43LS and L9LS. Recently, the first-in-human phase 1 clinical trial of TB31F in malaria-naïve healthy adults was reported [146]. TB31F was well tolerated with no safety concerns. A single 1 mg/kg intravenous dose was found to confer 80% transmission-reducing activity (TRA) against a West African *P. falciparum* strain. TRA was maintained for ≥84 days following a single intravenous 10 mg/kg dose [146]. Additionally, this regimen demonstrated >80% TRA against a genetically distant Asian *P. falciparum* strain for ≥84 days. Data modelling suggests that community-wide administration (at 80% coverage) of TB31F over a 3-year period could reduce clinical incidence of malaria by 54% (381 cases averted per 1000 people per year) in high-transmission settings, and by 74% (157 cases averted per 1000 people per year) in low-transmission settings [147]. It is proposed that annual or biannual administration of TB31F may be an effective intervention against malaria in seasonal transmission settings [147].

### 7.3. P. falciparum Erythrocytic mAbs

Erythrocytic malaria mAbs, those directed against parasitised erythrocytes, are yet to enter human trials. Slower progress in this domain is due to both biological differences (such as antigenic variation and immune evasion strategies) and the significant parasite burden at this life cycle stage, with a resultant increased risk of inducing resistance [136]. There are mAbs being investigated in preclinical studies that target apical membrane antigen 1 (AMA1, a protein essential for sporozoite and merozoite invasion); hmAb 75B10 (anti-AMA1 mAb) demonstrates potent strain-transcending neutralisation of *P. falciparum*, although enhancement of solubility, stability and delivery is needed [148].

### 7.4. mAbs Targeting P. vivax

There is a lack of mAbs against *P. vivax* malaria for reasons similar to why *P. vivax* vaccine development has been slow. However, one *P. vivax* mAb in the early stages of development, namely humAb826827, has shown particular promise [149]. This mAb, which primarily targets *P. vivax* AMA1 (PvAMA1), has demonstrated potent dual inhibitory activity towards *P. vivax* pre-erythrocytic and erythrocytic stages in an in vitro study of human hepatocytes and a murine model. Human trials are awaited should humAb826827 progress further.

### 7.5. Potential Approaches to the Use of mAbs

Compared to vaccines, mAbs offer several advantages, including rapid onset of protection and no reliance on host immunity [136]. There is the potential for their administration alongside the now available malaria vaccines to engender a more profound effect on malaria prevention in the longer term. A preclinical murine study demonstrated that administration of anti-PfCSP mAbs after administration of the *P. falciparum* R21 vaccine further enhanced protection [150]. However, the impact of this dual protective strategy in humans and at a population-based level is yet to be determined. The sequence and timings of dual administration are also key as there is theoretical risk that malaria mAbs could impair human host immune responses to malaria vaccines if the mAb is given prior to (and within the span of its half-life) or at the same time as the vaccine. This aspect will require further investigation if malaria mAb use is recommended in the future.

The WHO has set out priority groups in which mAbs might be used. These include vulnerable populations in which interventions are falling short of target due to poor adherence. A single mAb dose has the potential to replace the currently required multiple doses (typically 3–4 doses) of oral antimalarial drugs in SMC [135]. mAbs could also be used as an alternative to mass drug administration or drug-based prophylaxis for high-risk travellers or migrant workers [150]. Exploring their potential for use in travellers to malaria-endemic regions is important, particularly given that adherence to chemoprophylaxis remains a major concern [151]. mAbs could help overcome compliance barriers, providing a practical alternative to tablet-based prophylaxis. Immunocompromised individuals, who may mount suboptimal immune responses to malaria vaccines, are another group who could particularly benefit from malaria mAbs [152].

Malaria mAbs represent a promising future adjunct in malaria prevention, offering rapid protection without dependence on host immune priming. As mAbs act through passive immunisation, resulting in protection that is significantly more shorter-lived than an acquired immune response, technical advances are needed before large-scale deployment of mAbs in order to optimise their biophysical properties (including modifications to improve half-life and Fc effector function) as well as overall reductions in manufacturing costs [135]. An additional potential limitation to the widespread use of malaria mAbs is the emergence of resistance, although future development of mAbs with multiple antigenic targets (directed at differing stages of the *Plasmodium* life cycle) could reduce this risk [136]. The development of multi-target malaria mAbs, however, poses further technical and financial challenges.

## 8. Community Engagement in Malaria Prevention Approaches

Community beliefs regarding the causation and preventability of malaria largely determine engagement with, and therefore impact of, prevention tools and services [153]. Malaria prevention efforts often fail when they are designed and implemented without a deep understanding of the social and cultural context in which they operate [153]. Community engagement (CE) is therefore essential for culturally aligned, acceptable, and sustainable malaria prevention strategies [154].

Current efforts to strengthen community engagement in malaria programmes focus largely on health education. Many countries have adopted community health workers (CHWs), volunteers, teachers, religious leaders, and local youth groups to deliver culturally tailored health education [154]. For example, in Ebonyi State, Nigeria, female community volunteers, selected by village leaders, undergo structured training with the aim of these volunteers serving as health advocates embedded within their communities [155]. In Cambodia, Bangladesh and Malaysia, arts-based and culturally resonant communication methods have been deployed to reach populations who would not typically be receptive to formal health messaging [156].

CHWs are fundamental to bridging communities and healthcare initiatives, especially if the CHW is a trusted local resident [157]. For example, scaling-up of the Accredited Social Health Activists (ASHAs) programme in Odisha, India, has proven effective in reducing malaria transmission. ASHAs conducted fever screening, Rapid Diagnostic Test (RDT) testing, treatment follow-up, LLIN distribution and promotion, and IRS promotion in the region, contributing to a 94% decline in malaria cases between 2016 and 2021 [156]. A consistent presence of CHWs is essential to build trust and be truly effective, which is frequently undermined by the disjointed hiring of CHWs for short-term or seasonal intervention work [157]. As a sustainable alternative, in Myanmar, the expansion of the CHW role beyond ‘malaria-only’ in low-transmission areas to provide a broader package of basic healthcare services alongside proved effective in maintaining community uptake of malaria prevention [157].

Community-led and co-designed interventions are often successful in expanding malaria prevention efforts as they are more closely aligned to the community’s ethos and needs. In Laos, a five-step community engagement model, underpinned by shared leadership, was implemented with the aim of increasing MDA coverage in low-literacy remote villages [156]. This proved effective, achieving over 85% MDA coverage [156]. Community-driven initiatives have been particularly effective in promoting engagement with vector control programmes [158]. For example, in Indonesia, the ‘3M/3M+’ and ‘Jumantik’ schemes strengthened local ownership and improved larval source management [156]. Buy-in from residents leads to more accurate breeding site identification, greater coverage (as many breeding sites are on private land and household compounds), and reduced dependence on external teams (reducing costs and boosting sustainability) [158].

Highly mobile, hard-to-reach communities, such as the Artisanal and Small-Scale Gold Mining (ASGM) population in the Guiana Shield, northeast South America, pose a great challenge to malaria prevention efforts, as individuals are often transboundary and undocumented, with limited access to formal healthcare [159]. In order to address this, the Curema project in the Guiana Shield, which encompasses Guyana, Suriname, French Guiana, Venezuela and small parts of Colombia and northern Brazil, consisted of a bottom-up approach, incorporating CHWs, self-testing ‘Malakits’ and co-designed culturally adapted Information, Education, Communication (IEC) strategies [159].

Despite the illustrated positive impact of effective CE initiatives, gaps persist. In many endemic regions, sociocultural barriers such as traditional beliefs concerning gender norms and disease causality, layered with mistrust of healthcare bodies, restrict engagement with malaria prevention initiatives [154]. Amongst some African communities, malaria is still attributed to witchcraft or spiritual causes, such as ‘degedege’, reducing perceived severity and thus undermining uptake of vaccines and other prevention [153,160]. Furthermore, there is also a declining perceived risk of malaria in low-transmission settings, especially amongst younger people, reducing motivation to participate in elimination efforts [157]. In addition to sociocultural barriers, those of systemic and structural origin have limited the effectiveness of CE initiatives. For example, there is often poor continuity in prevention efforts due to high CHW turnover, weak supervision and inconsistent supply chains [154].

Many CE programmes foster shallow levels of engagement, focusing only on the foundational levels of informing and involving communities, failing to progress to a balanced collaboration or co-leadership [154]. Unlike the current status quo, to be truly effective CE should be iterative and proactive, with early involvement and frequent feedback [157]. A genuine shift to consulting, collaborating, and co-leading, with a focus on guidance and support from community leadership during local strategy development, could strengthen CE efforts [154,157]. In-depth socio-behavioural research to understand local perceptions, fears, and priorities supports the creation of effective IEC materials to tackle persisting misconceptions [160]. This is particularly important in the context of malaria vaccinations in the post-pandemic era in which the spreading of misinformation and conspiracy theories during the rollout of COVID-19 vaccinations has compounded existing hesitancy [159,160]. It is also recommended that malaria prevention efforts are integrated into broader community priorities to increase relevance and participation [159].

Overall, CE improves coverage, adherence, and sustainability across interventions [154]. This is achieved through enhancing cost effectiveness by leveraging local labour, reducing reliance on external teams, and improving access to private land for vector control, while strengthening a sense of ownership, which is critical for long-term elimination [157,158]. Ultimately, CE is key to successful adoption and longevity of malaria prevention strategies.

## 9. Conclusions

Effective future malaria prevention necessitates a comprehensive, multilayered strategy that leverages both established and novel interventions. The recent WHO-recommended rollout of RTS,S/AS01 and R21/Matrix-M vaccines represents a pivotal advancement in malaria prevention, but which requires effective integration with existing control measures. The emerging potential of malaria mAbs offers a promising additional prophylactic approach. Future success of malaria preventative measures and their sustained implementation hinge on ongoing financial investment, operational research tailored to localised transmission settings, and continuous parasite surveillance for emerging parasite resistance. This should be coupled to adaptive strategies that educate at-risk populations in order to overcome existing barriers to uptake.

## Data Availability

No new data were created or analysed in this study. Data sharing is not applicable to this article.

## Abbreviations

ASHA	Accredited Social Health Activist
ASGM	Artisanal and Small-Scale Gold Mining
CE	Community Engagement
CHW	Community Health Worker
DAI	Dual Active Ingredient
DDT	Dichlorodiphenyltrichloroethane
DEET	N,N-Diethyl-meta-toluamide
GMM	Genetically Modified Mosquitoes
IEC	Information, Education, and Communication
IPTp	Intermittent Preventive Treatment in pregnancy
IPTsc	Intermittent Preventive Treatment in school-aged children
IRS	Indoor Residual Spraying
ITN	Insecticide-Treated Net
LLIN	Long-Lasting Insecticidal Net
mAb	Monoclonal Antibody
MDA	Mass Drug Administration
MRP	Mass Relapse Prevention
MVIP	Malaria Vaccine Implementation Programme
PDMC	Post-Discharge Malaria Chemoprevention
PfCSP	*Plasmodium falciparum* Circumsporozoite Protein
PMC	Perennial Malaria Chemoprevention
PMD	p-Menthane-3,8-diol
PvCSP	*Plasmodium vivax* Circumsporozoite Protein
RDT	Rapid Diagnostic Test
RR	Rate Ratio
SAE	Severe Adverse Event
SMC	Seasonal Malaria Chemoprevention
SP	Sulfadoxine-Pyrimethamine
SPAQ	Sulfadoxine-Pyrimethamine plus Amodiaquine
TBV	Transmission-Blocking Vaccine
USEPA	United States Environmental Protection Agency
WHO	World Health Organization
